# Subliminal Priming Effects of Masked Social Hierarchies During a Categorization Task: An Event-Related Brain Potentials Study

**DOI:** 10.3389/fnhum.2022.862359

**Published:** 2022-07-07

**Authors:** Sabela Fondevila, David Hernández-Gutiérrez, Javier Espuny, Laura Jimenez-Ortega, Pilar Casado, Francisco Muñoz Muñoz, José Sánchez-García, Manuel Martín-Loeches

**Affiliations:** ^1^Center UCM-ISCIII for Human Evolution and Behavior, Madrid, Spain; ^2^Departamento de Psicobiología y Metodología en Ciencias del Comportamiento, Universidad Complutense de Madrid, Madrid, Spain; ^3^Basque Center On Cognition, Brain and Language – BCBL, Donostia-San Sebastian, Spain

**Keywords:** social hierarchy, masked priming, object processing, event related-brain potentials, N1, P3

## Abstract

Evidence so far shows that status detection increases attentional resources, especially for high hierarchies. However, little is known about the effects of masked social status cues on cognition. Here, we explore the masked priming effects of social status cues during a categorization task. For this purpose, we use Event-Related brain Potentials (ERP) time-locked to the presentation of two types of artworks (Christian, non-Christian) primed by masked social hierarchies sorted into two types (religious, military), and in two ranks (high, low) each. ERP results indicate early attention effects at N1, showing larger amplitudes for the processing of artworks after high and military ranks. Thereafter, the P3a increased for all artworks primed by religious vs. military figures, indicating a relevant role of task demands at this processing stage. Our results remark the automaticity of hierarchy detection and extend previous findings on the effects of social status cues on complex cognitive processes.

## Introduction

Status is a key social factor with deep evolutionary roots (Cummins, 2005). Status ranks individuals and distinguishes leaders from subordinates, conferring different levels of privileges and prestige (Fiske et al., 2016). Hence, being aware of one’s own status and the status of others facilitates adaptive behaviors in a group (Sapolsky, 2004). Individuals detect status rapidly and effectively even in the absence of awareness (Fondevila et al., 2021). In this regard, it has been shown that people can automatically infer status from certain cues (Moors and De Houwer, 2005; Zitek and Tiedens, 2012). For example, characteristics such as gender, age, race, or body/head postures convey social status (Fiske, 2010). Other non-verbal communicative features like body/head postures or gaze direction can also evoke status (Hall et al., 2005; Foulsham et al., 2010). Further, some symbols can strongly denote information about social hierarchy. For instance, pedestrians go against traffic signals more often when following jaywalkers dressed in suits and ties (Guéguen and Pichot, 2001), and drivers show more respect and less aggressive behavior toward high-status vehicles (Stephens and Groeger, 2014). In this regard, Chiao et al. (2004) presented a set of insignias for all the ranks within the US Navy Commissioned Officer System to investigate the mental representation of social status. Results indicated that US Navy insignias can mediate the processing of status perception to rapidly produce an internal representation of its semantic meaning.

Some neuroimaging studies have explored the neural correlates of this automatic detection of status by means of symbolic cues. The presentation of ranked stimuli, for instance, a series of numbers (non-social symbols) and US Navy insignias (social symbols), activated overlapped brain regions within the inferior parietal cortex (IPC) (Chiao et al., 2009) and prefrontal cortex (Zink et al., 2008), suggesting a core neural substrate for the processing of rank. By implementing social status through head postures, occupations, or status-face associations with a star system (i.e., three stars denoting high ranks and one star denoting low rank), studies using Event-Related brain Potentials (ERP) have demonstrated automatic effects of social status on both early (Arviv et al., 2015; Feng et al., 2015; Santamaría-García et al., 2015) and late (Breton et al., 2014, 2019; Furley et al., 2017) attentional and motivational processes. Taken together, these studies suggest that higher hierarchies typically enhance attention, showing larger amplitudes for attention-related ERP components (i.e., N170, N1, P3, LPP) than lower hierarchies. Further, social status also modulates decision making (Santamaría-García et al., 2014), social evaluation processes (Gyurovski et al., 2017), or social mindfulness judgments (Lu et al., 2020).

Though the effects of status perception have been a matter of interest in social neuroscience research, its unconscious processing has been almost unexplored. In a previous ERP study by our group, we used masked military figures as primes of a subsequent flanker task-inducing error. The ERN (error-related negativity), an index of error processing, was larger after the presentation of high relative to low social ranks, suggesting that higher hierarchies increase error processing by boosting attentional control to perform the ongoing task (Fondevila et al., 2021). To extend these findings, we here explore the subliminal priming effects of social status cues during a visual categorization task. This paradigm has been broadly used to assess the influence of a masked stimulus on the processing of a subsequent target (Van den Bussche et al., 2009; Kiefer et al., 2012). To explain the nature of this influence, the importance of top-down control processes has been raised. According to the *attentional sensitization model* by Kiefer and Martens (2010), top-down factors such as task sets (i.e., task content and demands) sensitize attention during subliminal priming. That is, task sets may enhance attention to unconscious stimuli when they are congruent while attenuating the processing of task-irrelevant (incongruent) unconscious stimuli. As a result, task sets originate top-down signals from the prefrontal cortex that monitor processing by increasing or decreasing attention to task congruent or incongruent pathways, respectively (Kiefer et al., 2012). Recently, two related studies have employed this methodological approach to investigate the effects of status on cognition and behavior (Boukarras et al., 2020, 2021). In a first study, Boukarras et al. (2020) explored the effects of social status figures (primes) on aesthetic judgments (pleasantness ratings) of abstract stimuli (Chinese ideograms), and they observed that ratings were significantly higher after the high-rank primes relative to the low-rank ones. This result showed, for the first time, that an explicit evaluation can be modulated by an implicit preference for social status attribution. In a second study, the same authors observed modulations of coordinated movements by the implicit attitudes (preferences) toward the interaction partner, which could be of high or low rank (Boukarras et al., 2021). Importantly, these studies demonstrated implicit effects of social status on performance measurements using a subliminal priming paradigm. In this regard, the present study joins this line by exploring the time course of subliminal social status effects during a categorization task, using ERPs.

At the electrophysiological level, certain ERP components have been shown to be modulated by attention and task demands in subliminal priming paradigms (for a review see Elgendi et al., 2018). Of particular interest for our purposes, the N1 ERP component is a negative deflection peaking at around 100 ms in posterior brain sites after the stimulus onset (Di Russo et al., 2002), which presumably reflects discriminative processes between attended stimuli (Vogel and Luck, 2000). Larger amplitudes of N1 would indicate enhanced attentional processing of stimulus features for discrimination purposes. Additionally, the P3 is a positive wave between 250 and 500 ms that reflects the automatic allocation of attentional resources and, importantly, it is highly sensitive to task demands (Polich, 2003, 2007). The ERP literature has distinguished between a fronto-central P3a and a more parietal P3b component. The former would be driven by frontal attention mechanisms during task processing, whereas the latter would reflect attention and memory processing along with temporoparietal areas (Polich, 2007).

In the present work, we explored the subliminal priming effects of masked social status figures during a categorization task, while recording ERP. To investigate social rank effects, we chose two types of well-established, recognized, and highly hierarchical social cues as priming subliminal stimuli: high and low rank religious and military figures. Military and religious sectors denote rank through their attires which typically have signed with symbolic well-known rank meanings. Then, to investigate the priming effects of those religious (Christian) and military (non-Christian) symbolic cues, participants performed a categorization task over a set of both religious (Christian) and non-religious (non-Christian) artworks. Using this design, we can explore whether the symbolic meaning of religious (and military) signs could specifically affect the categorization and processing of artworks congruent or not (religious-Christian and non-Christian, respectively) with the symbolic meaning of the primes. First, we predict social rank priming effects on attention; thus, early attention-related ERP components such as N1 would show larger amplitudes for artworks primed by high-status figures relative to those primed by low-status ones. Second, we predict top-down priming effects of task sets at P3, according to the attentional sensitization model for subliminal priming processing. In this regard, the categorization task (i.e., discriminating between Christian and non-Christian artworks) would enhance attention resources for subliminal religious (Christian primes), which in turn may modulate stimulus discrimination and task performance. Accordingly, the P3 amplitude should be larger for Christian artworks when primed by religious-Christian social cues in comparison to non-Christian military ones.

## Materials and Methods

### Participants

Twenty-four undergraduate students (12 females, 12 males) ranging from 19 to 29 years of age (mean = 25.7 years) participated in this experiment. They had a normal or corrected-to-normal vision and had no documented history of neural or psychiatric disorders. We carried out the study according to the Declaration of Helsinki and was approved by the ethics committee of the Center UCM-ISCIII for Human Evolution and Behavior. All the participants grew up in Spain, a traditionally Catholic community, and, thus, they all were familiar with Christian culture and symbols.

### Stimuli

**FIGURE 1:**
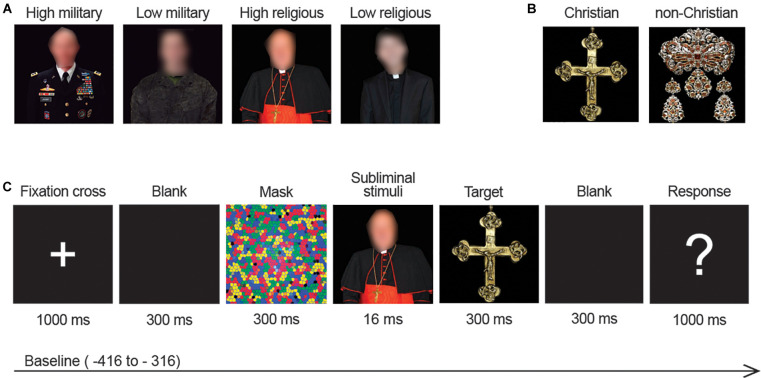
**(A)** Examples of the subliminal primes (from left to right): a colonel (military type, high rank), a soldier (military type, low rank), a cardinal (religious type, high rank), and a priest (religious type, low rank). **(B)** Examples of target stimuli: Christian artwork (left) and non-Christian artwork (right). **(C)** Experimental procedure.

Experimental stimuli consisted of a set of 720 images of objects (artworks), 4 social hierarchy figures of different types and ranks, and a mask (see Figures 1A,B). For experimental stimuli validation and preparation (see Supplementary Material).

### Procedure

All stimuli were presented at the center of an LCD screen (60 Hz refresh) controlled by Presentation^®^ Software (Neurobehavioral Systems). Participants’ eyes were 65 cm away from the screen, yielding a visual angle of 8.8° either vertically or horizontally in all objects.

Participants were seated in a sound-attenuated, electrically shielded room. They were informed about the categorization task and received instructions to press either one among two buttons in the response box, depending on whether they judged an artwork as Christian or non-Christian. The session started with a training phase of 30 trials followed by the experimental phase of 720 trials. Each trial began with a fixation cross of 1,000 ms. Thereafter, a blank screen of 300 ms, a forward mask (300 ms), and a hierarchical figure prime (16, 67 ms) was presented. Immediately afterward, the target stimuli (artwork picture) appeared for 300 ms, followed by a blank screen for 300 ms, and finally a question mark of 1,000 ms duration that requested the participant to respond to the categorization task (see Supplementary Material for the experimental procedure). Participants were instructed to fix their gaze on the cross and its location, and to refrain from blinking as much as possible (see Figure 1C for experimental procedure).

From the total of 720 artwork pictures (240 Christian, 240 non-Christian, 240 fillers) and the 4 social hierarchical primes of two status ranks and status types, we created four sets. Within every set, 60 artwork pictures of each type were primed by a picture of a cardinal (religious-Christian type, high social status), 60 by a priest (religious-Christian type, low social status), 60 by a colonel (military type, high social status) and 60 by a soldier (military type, low social status). Both primes and artworks presentations were randomized. We assigned the same number of participants (*n* = 6) to each of the four sets and allowed three short breaks during the experimental session.

### Post-task (Watching)

To ensure that the participants were not aware of the masked stimuli, they performed a *visibility task* immediately after the ERP recording session. The visibility task consisted of 36 trials that were identical to the trial sequence in the main experiment, except that there was no categorization task. That is, participants were presented with the masked social status figures followed by the artworks but, in this case, they did not have to categorize the Christianity of the images. Instead, they were asked to respond whether they had detected anything apart from the mask or the artwork image (a subjective measure of visibility, Ramsøy and Overgaard, 2004). In case they reported seeing something, participants had to communicate to the experimenter what they saw. After the 36 trials, none of them claimed to see neither the prime nor any other image apart from the mask and the artworks.

### EEG Recording and Pre-processing

EEG activity was recorded from 59 Ag/AgCl electrodes embedded in an electrode cap (Easycap^®^) using the extended 10–20 system. All scalp electrodes were referenced online to the right mastoid—M2—and re-referenced offline to the average of the left and right mastoids. Bipolar vertical and horizontal electrooculograms—EOG—were recorded for monitoring eye-related activity. Electrode impedances were kept below 5 kΩ. An online bandpass filter of 0.01–100 Hz was applied. Recordings were continuously digitized at a sampling rate of 250 Hz for the entire session. The continuous recording was divided into 1,416-ms epochs for each trial, with a baseline of 100 ms during the blank screen before the onset of the masked stimulus (from –416 to –316 ms relative to the target stimulus). Ocular artifacts were removed using Independent Component Analysis (ICA, Jung et al., 2000), implemented in Brain Vision Analyzer^®^ software. Following the ICA-based correction, we performed artifact rejection by visual inspection to discard those epochs still presenting artifacts. Trials with response omissions, incorrect categorizations, and RT outliers were also removed (see below). On average, 13.86% of the epochs were rejected, leading to an average of 51.13 trials per condition.

### Data Analysis

Data analyses were performed using the SPSS software package (Version 15.0; SPSS Inc., Chicago, United States). Behavioral and ERP data were analyzed using repeated-measures ANOVAs including a 2 × 2 × 2 factorial design: Image Type (two levels: Christian, non-Christian), (*Status*) prime Rank (two levels: high, low), (*Status*) prime Type (two levels: religious, military) as within-subject factors. The Greenhouse-Geisser (GG) epsilon correction was applied to adjust the degrees of freedom of the F-ratios when necessary, and *post hoc* comparisons to determine the significance of pair-wise contrast were corrected by the Bonferroni procedure. Effect sizes were computed using the partial eta-square (η^2^*_*p*_*) method.

### Behavioral Analysis

We analyzed the percentage of accuracy judgments (Christian images categorized as Christian and non-Christian images categorized as non-Christian) and RTs in the categorization task. A repeated-measures ANOVA on each behavioral measure was carried out. In the case of RTs, we excluded from the analysis outliers, defined as responses below 100 ms (Luce, 1986) and above 1,500 ms, which exceeded the response time given to participants.

### Event-Related Brain Potentials Analysis

**FIGURE 2:**
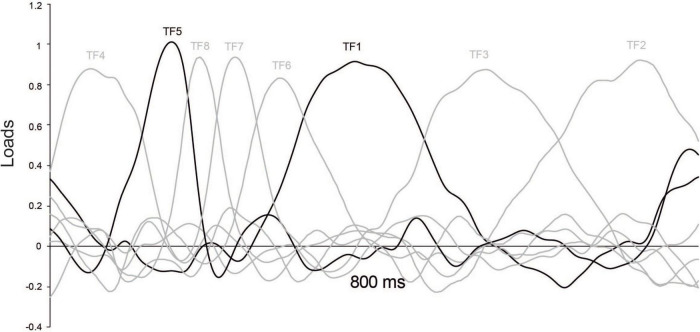
tPCA: factor loadings after Promax rotation. Temporal factors 5 (N1) and 1 (P3) are depicted in black.

To reliably test whether N1 and P3 were present in the ERPs, components explaining most of the variance in the temporal domain were detected and quantified through a covariance-matrix-based temporal principal components analysis (tPCA). tPCA is a valuable data-driven method to differentiate components over time, since it presents each ERP component with its “clean” shape, extracting and quantifying it free of the influences of adjacent or latent components. In brief, tPCA computes covariance between all ERP time points, which tends to be high between those time points involved in the same component and low between those belonging to different components (for a more complete description of PCA, see Carretié et al., 2004; Dien, 2012). The solution is a set of factors made up of highly covarying time points, which ideally correspond to separate, non-overlapped ERP components. The *Temporal factor score*, a tPCA-derived parameter that quantifies extracted temporal factors, is linearly related to amplitude. In the present study, tPCA was computed for 200 digitalized voltage points (from 0 to 800 ms) as data matrix variables, and 11,328 cases were obtained from the product of participants (24), conditions (8), and electrodes (59). The number of factors was selected based on a screen test (Cliff, 1987), and those extracted factors were submitted to Promax rotation (Dien, 2010), confirming the presence of N1 and P3 (see Figure 2).

Once quantified in temporal terms, the temporal factor scores were submitted to spatial PCA (sPCA) which “separates” ERP components based on space criteria. That is, each spatial factor ideally reflects one of the concurrent neural processes underlying each temporal factor. In the present experiment, sPCA decomposed the N1 and P3 topography at the scalp level into their main spatial regions. This spatial decomposition is an advisable strategy before statistical contrasts since ERP components frequently behave differently in some scalp areas than they do in others (e.g., they present opposite polarity or react differently to experimental manipulations). Each region or spatial factor is formed with the scalp points where recordings tend to covary. As a result, the shape of the sPCA-configured regions is functionally based. Remarkably, each spatial factor can be also quantified through the *spatial factor score*, a single parameter that reflects the amplitude of the whole spatial factor. We selected the number of spatial factors based on a screen test and subsequently submitted it to Promax rotation. In the case of sPCA, dependent variables were temporal factor scores at each electrode (59), and cases were participants (24) X conditions (8).

## Results

### Behavioral Data

Repeated-measures ANOVA showed an Image Type main effect on accuracy [*F*(1, 23) = 10.26, *p* < 0.01, η^2^*_*p*_* = 0.309] indicating higher accuracy ratings for non-Christian relative to Christian images. The remaining behavioral measures did not yield significant results (all *ps* > 0.05) (see Table 1 for behavioral results).

**TABLE 1:** Mean percentage of accuracy judgments (with mean values in parenthesis) and reaction times (RTs) during the categorization task for all conditions.

	Christian objects				Non-Christian objects			
	High Military	Low Military	High Religious	Low Religious	High Military	Low Military	High Religious	Low religious
Accuracy (%)	77.48 (46.79)	81.58 (48.96)	79.43 (47.67)	79.65 (47.79)	88.46 (53.08)	89.23 (53.54)	87.71 (52.63)	88.4 (853.04)
RTs (ms)	288.12	283.71	281.5	287.84	285.27	284.01	294.98	281.79

### Event-Related Brain Potentials Data

The tPCA applied to the ERP yielded a factorial structure consisting of eight main factors. Based on latencies and topographies, the correspondence between tPCA factors and ERP components for priming effects were: Factor 5 (peaking at 148 ms) related to the N1 waveform, and Factor 1 (peaking at 372 ms) related to the P3 waveform. The sPCA was subsequently applied to temporal factor scores and extracted three spatial factors for each component (N1 and P3). Image Type, Status Rank, and Status Type factors underwent separated repeated-measures ANOVAs on N1 and P3 spatial factor scores. All statistical results can be seen in Table 2. In the following only significant results (*p* < 0.05) are reported.

**TABLE 2:** Repeated measures ANOVAs for each spatial factor (SF: Anterior, Centro-Parietal, Posterior) of the two temporal factors (TF: N1, and P3).

TF	SF	SR (*df* = 1.23)	ST (*df* = 1.23)	IT (*df* = 1.23)	SR × IT (*df* = 2.46)	ST × IT (*df* = 2.46)	SR × ST (*df* = 2.46)	SR × ST X IT (*df* = 2.46)
**TF5 (N1)148 ms**	Anterior	*F* = 85, n.s.	*F* = 5.78* η^2^*p* = 0.20	*F* = 2.34, n.s.	*F* = 0.02, n.s.	*F* = 0.61, n.s.	*F* = 0.95, n.s.	*F* = 0.16, n.s.
	Centro-Parietal	*F* = 4.05* η^2^*p* = 0.15	*F* = 15.73*** η^2^*p* = 0.40	*F* = 2.09, n.s.	*F* = 0.03, n.s.	*F* = 0.11, n.s.	*F* = 0.05, n.s.	*F* = 0.003, n.s.
	Posterior	*F* = 2.14, n.s.	*F* = 0.19, n.s.	*F* = 0.67, n.s.	*F* = 0.15, n.s.	*F* = 0.94, n.s.	*F* = 0.001, n.s.	*F* = 0.37, n.s.
**TF1 (P3)372 ms**	Anterior (**P3a**)	*F* = 3.02, n.s.	*F* = 4.54* η^2^*p* = 0.16	*F* = 20.17*** η^2^*p* = 0.46	*F* = 0.72, n.s.	*F* = 2.02, n.s.	*F* = 1.52, n.s.	*F* = 0.03, n.s.
	Centro-Parietal	*F* = 0.32, n.s.	*F* = 0.40, n.s.	*F* = 9.98** η^2^*p* = 0.30	*F* = 0.75, n.s.	*F* = 0.35, n.s.	*F* = 0.91, n.s.	*F* = 0.03, n.s.
	Posterior	*F* = 0.02, n.s.	*F* = 3.54, n.s.	*F* = 4.36* η^2^*p* = 0.16	*F* = 0.04, n.s.	*F* = 0.07, n.s.	*F* = 0.96, n.s.	*F* = 2.04, n.s.

Effect sizes of significant results were all above 0.15 (η^2^_p_ > 0.15). SR, Status Rank; ST, Status Type; IT, Image Type; df, degrees of freedom; n.s., non-significant, *p < 0.05, **p < 0.01, ***p < 0.001.

**FIGURE 3:**
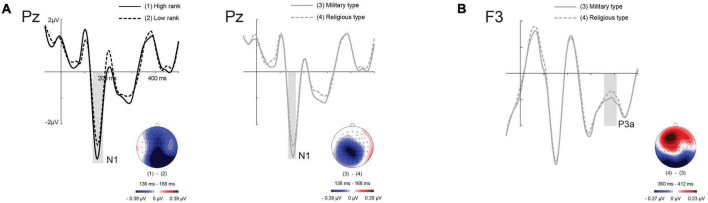
Grand averages target-locked for **(A)** status rank (left) and status type (right) effects in N1 at Pz channel; **(B)** status type effect in P3a at F3 channel.

**N1:** The centroparietal N1 spatial factor yielded a significant main effect of Status Rank [*F*(1, 23) = 4.05, *p* < 0.05, η^2^*p* = 0.15], with larger amplitudes for artworks primed by the high in comparison to the low-rank figures. Also, a significant main effect of Status Type [*F*(1, 23) = 15, 73, *p* < 0.001, η^2^*p* = 0.4] indicated greater amplitudes for artworks primed by military relative to religious cues for both the centroparietal and the anterior spatial factors (see Figure 3A).

**P3:** Results showed a main effect of Status Type for the anterior (frontocentral) P3 spatial factor (P3a) [*F*(1, 23) = 4.54, *p* < 0.05, η^2^*p* = 0.16], showing greater amplitudes for artworks primed by Religious vs. Military figures. In addition, a main effect of Image Type was found to be significant for the three spatial factors; Anterior, Centro-parietal, Posterior [(*F*(1, 23) = 20, 17, *p* < 0.001, η^2^*p* = 0.46], [*F*(1, 23) = 9, 98, *p* < 0.01, η^2^*p* = 0.30], [*F*(1, 23) = 4.36, *p* < 0.05, η^2^*p* = 0.16, respectively] of the P3, showing larger amplitudes for Christian relative to non-Christian artwork pictures (see Figure 3B).

## Discussion

In this study, we explored the subliminal priming effects of social status masked cues during a categorization task. To this aim, we presented masked social ranked figures; two Christian (cardinal-high rank, priest-low rank) and two non-Christian (coronel- high rank, soldier-low rank), while recording ERP to artwork figures. Participants performed a categorization task, judging whether each artwork was either Christian (religious) or non-Christian (non-religious). Through this procedure, we explored the automaticity of hierarchy detection and the extent to which this detection could affect a subsequent categorization task. At the behavioral level, we only observed a main effect of Image Type on accuracy judgments, with higher ratings for non-Christian in comparison to Christian artworks. This suggests that, overall, participants found it slightly easier to identify non-Christian than Christian artworks, while the subliminal presentation of social status cues did not seem to affect the categorization processes. Importantly for this result, some studies have previously used a subliminal priming paradigm to investigate the effects of social status on performance (Boukarras et al., 2020, 2021). Specifically, one of them (Boukarras et al., 2020) used short (17 ms) and long (75 ms) presentation times for status primes and only found effects on the following task during the long condition. The authors pointed out that 17 ms could not be enough to extract social status knowledge and that a longer processing time is required to influence performance. However, in a previous study (Fondevila et al., 2021) we presented status primes for 16 ms and found subliminal effects on the subsequent task, showing that social status can be automatically processed using short presentations. A possible explanation for these discrepant results is that task complexity is playing a crucial role. In both the current study and that by Boukarras et al. (2020), participants performed complex decisions such as aesthetic judgments or artwork categorization, respectively, which could require sufficient cognitive resources to rule out the impact of subliminal status cues. Instead, in our previous work (Fondevila et al., 2021) participants performed a flanker task that did not involve a complex evaluative process and had no semantic links with any social information. Although possible, this interpretation is only tentative. So far, only these three studies have approached this topic and further research is needed to expand our understanding of how the subliminal processing of social hierarchies affects cognition and behavior.

At the electrophysiological level, we observed priming effects on attention by masked social ranked figures during the processing of artworks. As predicted, we found a main effect of Status Rank at the N1 latency, showing larger amplitudes for artworks primed by high- relative to low-ranked figures. This may indicate that subliminal high-ranked figures induced an increment of selective attention to better discriminate artworks’ features (Vogel and Luck, 2000; Di Russo et al., 2002). In the same line, other authors have found larger N1 amplitudes during a perceptual task after the presentation of high-ranked figures (Santamaría-García et al., 2014). Early attention modulations triggered by high-status cues are a common finding in electrophysiology research (Breton et al., 2014; Arviv et al., 2015; Feng et al., 2015). As a novelty, we observed this effect during a subliminal priming paradigm, emphasizing the automaticity of social hierarchies’ detection and extending previous findings of our group (Fondevila et al., 2021). Unexpectedly, the main effect of Status Type at N1 showed greater amplitudes during the processing of artworks after military relative to religious (Christian) masked primes at both centroparietal and anterior brain sites. This would suggest that priming effects linked to task sets (i.e., categorization of Christian and non-Christian artworks) did not affect early attention processing stages. Instead, this result may reflect that military symbols might have a greater social impact than religious symbols to evoke an early representation of ranked meanings (Chiao et al., 2004, 2009). Although this early hierarchy type distinction requires further investigations this finding highlights the N1 sensitivity to higher cognitive processes (Vogel and Luck, 2000; Wascher et al., 2009).

Our results also showed a main effect of Status Type at frontal P3 (P3a), with larger amplitudes for artworks primed by religious (Christian) relative to military figures (non-Christian). Then, religious (Christian) primes might have driven frontal attention mechanisms for artwork discrimination. Contrary to our predictions, religious (Christian) subliminal primes did not affect specifically Christian artworks processing. Instead, religious (Christian) primes enhanced attention to the whole set of artworks. Also, we observed a main effect of the Image Type factor for the three spatial factors of the P3 (anterior, centroparietal, and posterior), showing larger amplitudes for Christian relative to non-Christian artworks. This general P3 effect would indicate more attentional resources and memory processing activity when examining Christian objects for the subsequent categorization task. Behavioral accuracy results suggested that Christian artworks were more difficult to categorize properly than non-Christian ones. Regarding P3a findings, it is reasonable to infer that religious (Christian) primes triggered artworks evaluation engaging focal attention (P3a) to facilitate task demands (Polich, 2007). In this line, the *attentional sensitization model* (Kiefer and Martens, 2010) points out that task sets enhance attention to task-congruent pathways during subliminal priming paradigms. In our experiment, task sets (i.e., categorization of Christian and non-Christian artworks) might have influenced brain activity patterns boosting attentional resources to artworks primed by religious (Christian) figures, which in turn might have contributed to the categorization process. In a complementary way, the *P3 inhibition hypothesis* (Knight, 1997; Soltani and Knight, 2000) suggests that stimulus gaining attention automatically from task demands (Religious Christian primes) may impact focal attention, generating a frontal P3a by the anterior cingulate cortex and related structures. Then, attention-driven activity may be transmitted to temporoparietal areas where memory operations engage the P3b (Knight, 1997; Soltani and Knight, 2000). In this experiment, results showed that certain underlying processes of P3 were sensitive to top-down influences by task sets (P3a), while others remained unaffected by subliminal hierarchy primes (P3b). Although we failed to observe social rank effects at the P3 stage, the P3 is sensitive to discriminating between social hierarchy characteristics. For example, some authors have recently demonstrated different P3a frontal activations for moral vs. financial status (Gyurovski et al., 2017) or for legitimate vs. coercive hierarchies (Gangl et al., 2017). Again, these results suggest that electrophysiological signals modulated by task demands are highly sensitive to the detection and discrimination of complex social hierarchy features.

This study has some limitations to be considered. In the first place, social status effects are statistically not very strong, and we did not observe significant interactions. It is probably the case that increasing the number of participants might improve the statistical power. Also, the lack of significant behavioral results could indicate either that a longer presentation time is required for social status primes or the presence of an excessive cognitive load by task complexity (or both). However, a different experiment design is necessary to give a convincing explanation. To date, there are very few studies investigating the subliminal priming effects of social status cues. Thus, more research is needed to draw the general picture of how such complex social information affects cognition and behavior.

## Conclusion

Our results extend previous findings on the automaticity of social hierarchy detection. Using a subliminal priming paradigm, we explored how masked social hierarchies’ cues can affect subsequent cognitive operations, such as categorization processes. We observed significant effects at the electrophysiological level, suggesting changes in brain activity by well-recognized social hierarchies even in the absence of awareness, although our experimental manipulation did not affect performance. First, we observed that higher hierarchies specifically increase attentional resources during visual processing in a categorization task. Second, top-down influences seemed to enhance the processing of subliminal primes congruent to task demand, showing priming subliminal effects of social hierarchies for the first time. Overall, these findings reinforce evidence signaling how human cognition deals effectively with complex social information regardless of conscious awareness.

## Data Availability Statement

The datasets presented in this study can be found in online repositories. The names of the repository/repositories and accession number(s) can be found in the article/Supplementary Material.

## Ethics Statement

The studies involving human participants were reviewed and approved by the Ethics Committee of the Complutense University. The patients/participants provided their written informed consent to participate in this study.

## Author Contributions

SF, DH-G, JE, MM-L, LJ-O, PC, FM, and JS-G contributed to the conception and design of the study. SF, JE, and JS-G organized the database and performed the statistical analysis. SF wrote the first draft of the manuscript. All authors contributed to manuscript revision, read, and approved the submitted version.

## Conflict of Interest

The authors declare that the research was conducted in the absence of any commercial or financial relationships that could be construed as a potential conflict of interest.

## Publisher’s Note

All claims expressed in this article are solely those of the authors and do not necessarily represent those of their affiliated organizations, or those of the publisher, the editors and the reviewers. Any product that may be evaluated in this article, or claim that may be made by its manufacturer, is not guaranteed or endorsed by the publisher.
