# Supplementary material for: Subliminal Priming Effects of Masked Social Hierarchies During a Categorization Task: An Event-Related Brain Potentials Study
Source: Front Hum Neurosci. 2022 Jul 7;16:862359. doi: 10.3389/fnhum.2022.862359 (PMC9301232; doi:10.3389/fnhum.2022.862359)
Supplement: Supplementary file 1 [file Data_Sheet_1.docx]

**Masked priming effects of social hierarchies during a categorization task: An ERP study**

**Supplementary Materials**

Contents

1- Stimuli (artworks) validation and preparation (norming study).

2- Social hierarchy figures (subliminal stimuli) and mask.

3- Behavioral Results.

1. **Stimuli (artworks) validation and preparation**

First, we collected an original set of 1917 stimuli from the Victoria and Albert Museum (2017) web database, which classifies artworks according to their provenance, material, technique and other relevant characteristics such as their religious content. Second, from this original set, 1243 (64%) were classified as Christian and had explicit Christian symbols: a Cross, an aureole, a Jesus or a Virgin Mary image, etc. The remaining pictures (36%) were non-Christian (nor actually belonged to any other religion) and displayed similar composition and overall arrangement as the Christian stimuli.

Third, we recruited two groups of participants to validate the stimuli (stimuli norming study). One was a Christian group that belonged to the University chapel (22 participants; 16 females, 6 males; age range = 19-24 years), and the other a non-Christian (non-religious) group who responded to an ad at the university noticeboard: “*looking for non-believers for a research project in psychology”* (21 participants; 18 females, 3 males; age range = 18-24 years). Both groups fulfilled a six-items religiosity questionnaire using a 10-point Likert scale ranging from 1 (*I do not agree at all*) to 10 (*I do totally agree*) to determine their level of agreement with each statement. The religiosity questionnaire and the item scores for each group is shown below (Table S1).

| Religiosity questionnaire | Christian: mean (SD) | non-Christian: mean (SD) |
| --- | --- | --- |
| 1. I believe in the existence of God | 9.76 (.46) | 1.18 (.46) |
| 2. I am a Christian person | 9.42 (1.29) | 1.13 (.30) |
| 3. My beliefs play a major role in decision making | 9.09 (.98) | 1.045 (.30) |
| 4. I attend mass regularly (at least one a week) | 9.28 (2.66) | 1.045 (0) |
| 5. I pray regularly | 8.9 (1.12) | 1.045 (0) |
| 6. I participate regularly in religious activities | 8.54 (2.58) | 1.22 (.82) |
| Total (mean) | 9.17 | 1.11 |

**Table S1**. Religiosity questionnaire scores and standard deviations (in parenthesis) for the Christian and the non-Christian samples.

Fourth, to avoid religious knowledge biases, both the Christian and the non-Christian groups rated the 1917 art objects set using a 5-point *Likert* scale (from 1 – *non-Christian or non-devotional* – *to* 5 – *absolutely Christian or devotional*). Stimuli appeared one by one with no time limit to respond, using Presentation^®^ Software. Thereafter we eliminated all objects with standard deviations (SD) > 1.66 in each group to limit the variability in the scores. We also eliminated stimuli with significant mean rating differences between the Christian and the non-Christian groups (*student’s t* comparisons p < .05). Then, those stimuli equally rated in the Christian and non-Christian groups, with means between 3.66 and 5, were labeled as Christian artworks, and those with means between 1 and 2.33 were labeled as non-Christian artworks. Finally, those with means between 2.33 and 3.66 were used as fillers. Mean scores and standard deviations (in parenthesis) for the validation of artworks in the Christian and non-Christian samples are shown in Table S2 below.

|  | Christian sample | | non-Christian sample | |
| --- | --- | --- | --- | --- |
| Type of artworks | Christian | Non-Christian | Christian | Non-Christian |
| Decorative objects | 4.34 (.81) | 1.72 (.96) | 4.37 (.83) | 1.84 (1.03) |
| Figures/sculptures | 4.27 (.85) | 1.66 (.88) | 4.35 (.91) | 1.72 (.97) |
| Reliefs | 4.17 (.93) | 1.75 (1.02) | 4.21 (1.05) | 1.88 (1.10) |
| Paintings | 4.05 (.69) | 1.62 (.89) | 4.57 (.71) | 1.75 (1.00) |
| Fabrics | 4.20 (.91) | 1.77 (1.03) | 4.29 (.98) | 1.88 (1.08) |
| Stained-glass | 4.28 (.90) | 1.88 (1.09) | 4.44 (.80) | 1.91 (1.13) |
| TOTAL | 4.31 (.84) | 1.73 (.97) | 4.37 (.86) | 1.83 (1.05) |

**Table S2**. Mean scores and standard deviations (in parenthesis) for the validation of artworks in the Christian and non-Christian samples. The final set included decorative objects, figures/sculptures, reliefs, paintings, fabrics, and stained glasses.

Fifth, we matched stimuli in visual aspects, that is, spatial location, luminance, and shape, as these may affect the early stages of sensory-perceptual processing (Johannes, Münte, Heinze & Mangun, 1995). We executed this as follows. Both the selection of Christian and non-Christian artworks included decorative objects, sculptures, reliefs, paintings, fabrics and stained-glass in comparable numbers and materials. We used Adobe Photoshop^®^ to set the background of all pictures into black (0, 0, 0 in the RGB scale). We also equalized the average luminance between the Christian (mean = 51.82 cd/m², SD = 31.70) and the non-Christian artworks (mean = 51.27 cd/m², SD = 34.04), with no significant differences (t = .209, *p* = .83). Finally, we centered and matched the size of all the objects to 400 x 400 pixels (i.e. same height or same width in the case of vertical or horizontal objects, respectively). Thus, we obtained a sample of pictures with high visual consistency in the classification of objects as Christian or non-Christian. Additionally, regardless of the religiosity of the participants, they were highly similar in social and cognitive variables to those in the ERP study. Examples of the artworks presented can be seen in Figure 1b.

1. **Social hierarchy figures (subliminal stimuli) and mask**

We selected four social hierarchy figures of two different types and ranks: a cardinal (religious-Christian type, high rank), a priest (religious-Christian type, low rank), a colonel (military type, high rank) and a soldier (military type, low rank). All four figures displayed their original attires, including their corresponding insignias of status, which have been probed to effectively evoke status (Chiao et al., 2004; Chiao et al., 2009). In addition, faces looked blurred to avoid processing effects of facial expressions or attributes. The four social hierarchy figures matched in visual aspects such as size (400 x 400 pixels) and shape. Their luminance values slightly varied because of the different insignias and attires corresponding to the symbolic cues denoting type and status. Luminance values were as follows: high religious-Christian (mean = 38.22 cd/m², SD = 46.19), low religious-Christian (mean = 28.401 cd/m², SD = 40.47), high military (mean = 29.42 cd/m², SD = 40.84), low military (mean = 22.46 cd/m², SD = 40.84) within a range from 0 to 255 (RGB scale)). We carried out statistical T-test comparisons of independent samples with 95% confidence intervals for the difference to examine these slightly different values. Neither the compassion between high and low religious primes (t=0.78, p=0.43) nor the main comparison between high and low military primes (t=0.59, p=0.55) yielded significant results. It is important to note for our experimental purposes that luminance frequently modulates early attention (Johannes et al., 1995; Manolas, Stamoulos, & Anninos, 1999). Previous studies have found luminance effects on early attention with differences ranging from 20 cd/m² and 50 cd/m² between conditions, even when stimuli were not attended (Johannes et al., 1995). In this experiment, differences in luminance within Status Type and Status Rank were lower than 8.5 cd/m² and 1.5 cd/m², respectively (33.31 cd/m² for religious, 25.94 cd/m² for military, 33.82 cd/m² for high-, and 25.43 cd/m² for low-rank subliminal figures) and more importantly, statistical analysis revealed non-significative differences between high and low primes. Thus, they should not be responsible for early attention modulations. Examples of social hierarchy figures can be seen in Figure 1a.

**References**

Chiao, J. Y., Bordeaux, A. R., & Ambady, N. (2004). Mental representations of social status*. Cognition, 93(2)*, B49-B57. DOI: 10.1016/j.cognition.2003.07.008

Chiao, J. Y., Harada, T., Oby, E. R., Li, Z., Parrish, T., & Bridge, D. J. (2009). Neural representations of social status hierarchy in human inferior parietal

cortex. *Neuropsychologia, 47(2)*, 354-363. DOI: 10.1016/j.neuropsychologia.2008.09.023

Johannes, S., Münte, T. F., Heinze, H. J., & Mangun, G. R. (1995). Luminance and spatial attention effects on early visual processing. *Cognitive Brain Research,*

*2(3)*, 189-205. DOI: 10.1016/0926-6410(95)90008-x

Manolas, M. G., Stamoulos, T. D., & Anninos, P. A. (1999). Differences in human visual evoked potentials during the perception of colour as revealed by

a bootstrap method to compare cortical activity. A prospective study. *Neuroscience letters, 270(1)*, 21-24. DOI: 10.1016/s0304-3940(99)00457-7

Victoria Albert Museum. (2017). Retrieved from <http://collections.vam.ac.uk/>.
